# SP-1-activated LINC01016 overexpression promotes gastric cancer invasion and metastasis through inhibiting EIF4A3-mediated MMP9 mRNA decay

**DOI:** 10.1038/s41419-024-07250-z

**Published:** 2025-01-29

**Authors:** Ying Sun, Hui Zhang, Duan-bo Shi, Peng Gao

**Affiliations:** 1 Department of Pathology, Qilu Hospital and School of Basic Medical Sciences Shandong University, Jinan, Shandong PR China; 2 Department of Medical Oncology, Qilu Hospital of Shandong University (Qingdao), Qingdao, Shandong China

**Keywords:** Oncogenes, Long non-coding RNAs

## Abstract

Long noncoding RNAs (lncRNAs) are key regulators during gastric cancer (GC) development and may be viable treatment targets. In the present study, we showed that the expression of the long intergenic noncoding RNA 01016 (LINC01016) is significantly higher in GC tissues with lymph node metastasis (LNM) than those without LNM. LINC01016 overexpression predicts a poorer relapse-free survival (RFS) and overall survival (OS). Furthermore, we found that LINC01016 is activated by transcriptional factor SP-1 and contributes to the overt promotion of cell migratory ability. EIF4A3 was identified as a binding partner of LINC01016 by RNA pull-down assay, mass spectrometry and western blot. We determined that LINC01016 can blocks the binding of EIF4A3 to MMP9 mRNA, thereby inhibiting EIF4A3-mediated nonsense-mediated RNA decay (NMD), increasing MMP9 mRNA level and protein expression levels to promote tumor progression. LINC01016 or LINC01016-mediated EIF4A3/MMP9 may be potential therapeutic targets for patients with GC.

## Introduction

Gastric cancer (GC) is responsible for over 968,000 new cases in 2022 and an estimated 660,000 deaths, making it the fifth most frequently diagnosed cancer and the fourth leading cause of cancer death worldwide [1]. Annual rates of increment are markedly elevated in Eastern Asia, particularly in low-income countries [2]. Patients with gastric cancer are usually diagnosed at an advanced stage, characterized by malignant proliferation, extensive invasion, lymph node metastasis and drug resistance, commonly presenting with a high mortality rate [3]. Therefore, deciphering tumorigenesis and progression of GC, will benefit the identification of novel diagnostic biomarkers and development of new therapeutic strategies.

Long noncoding RNAs (lncRNAs) are a class of newly discovered nonprotein- coding RNA molecules over 200 nucleotides in length and have been shown to play critical roles in tumorigenesis, including in gastric cancer (GC) [4, 5]. LncRNAs participate in various biological processes, such as chromatin interaction, transcription regulation, guiding protein-DNA interaction, and epigenetic regulation. For example, the complex formed by LINC02273 with hnRNPL protein activates the transcription of the oncogene AGR2 by increasing the levels of H3K4me3 and H3K27ac around its promoter region [6, 7]. In another study, UCA1 was found to function as an onco-lncRNA, promoting GC cell proliferation and migration and inhibiting GC cell apoptosis by repressing the antitumor miRNAs miR-26a [8]. Therefore, identifying key lncRNAs involved in GC progression is of great significance for understanding the pathogenesis of this disease. We found abnormal expression of LINC01016 in GC tissue through high-throughput sequencing (GEO, GSE72307). However, the effect of LINC01016 on tumor metastasis in GC remains unclear.

In this study, we demonstrated that LINC01016 levels were higher in GC tissues with LNM, and could predict poor relapse-free survival (RFS) and overall survival (OS) outcomes. LINC01016 promotes migration and invasion both in vitro and in vivo of GC by increasing MMP9 mRNA and protein levels. This function is mediated by EIF4A3, which is an important component of the exon junction complex (EJC) complex, in the RNA monitoring mechanism of nonsense-mediated RNA decay (NMD). These findings suggest that LINC01016 represents a novel indicator of poor prognosis in GC and could be a potential therapeutic target and diagnostic marker.

## Materials and methods

### Clinical samples

A total of 80 primary GC tissues were collected from patients, including 51 cases of GC tissues with LNM and 29 cases without LNM, who had undergone surgery before receiving chemotherapy or radiation therapy in Qilu Hospital of Shandong University and Weifang people’s hospital from 2012 to 2014. Our sample size estimation is based on http://powerandsamplesize.com/Website. Tumor stage was defined basing on the criteria of the 8th Edition of the AJCC Cancer Staging. Our research was approved by the Institute’s Research Ethics Committee of Shandong University (LL-201501017) and conducted in accordance with ethical guidelines of the World Medical Association Declaration of Helsinki. Written informed consent was obtained for all patient samples.

### Microarray analysis

10 GC patient samples were prepared for human lncRNA microarrays (Human 8 × 60 K LncRNA Microarray v2.0). In brief, RNA was extracted and purified using Trizol reagent (Invitrogen, Carlsbad, CA, USA). Then, each sample was amplified, labeled, and hybridized to the Arraystar (Rockville, MD). Following the washing steps, the arrays were scanned by the Agilent Scanner G2505B, and array images were analyzed using the Agilent Feature Extraction software (version 10.7.3.1). Quantile normalization and subsequent data processing were performed using the GeneSpring GX v11.5.1 software (Agilent Technologies). Volcano plot filtering was employed to identify the lncRNAs with statistically significant differences and the threshold to screen upregulated or downregulated lncRNAs was identified at a fold change ≥ 2 and a *p*-value ≤ 0.05.

### Cell lines and culture

Human GC cell lines HGC27, BGC823, MKN45, MGC803 and human normal gastric epithelial cell line GES-1 were obtained from ATCC. Cells were cultured in 1640 medium (Gibco, Carlsbad, CA, USA) supplemented with 10% fetal bovine serum (FBS) (Gibco, NY, USA) in a humidified incubator under a 5% CO_2_ atmosphere at 37 °C.

### Cell transfection

For in vitro assays, three Antisense oligonucleotide (ASO, si-LINC01016-1, si-LINC01016-2 and si-LINC01016-3) targeting LINC01016 and negative control (si-NC) were designed and synthesized by RiboBio (Guangzhou, China). Only si-LINC01016-3 successfully knocked down LINC01016 for subsequent testing. Cells were transfected with ASO targeting LINC01016 using Roche X tremegene™siRNA transfection reagent. To overexpress LINC01016, human fulllength LINC01016 cDNA was amplified using PCR methods. The PCR products were ascertained by direct DNA sequencing and subcloned into the pcDNA3.1(+) vector (Invitrogen) between the NheI and NotI restriction sites. Plasmids were transiently transfected into GC cells with tuborfect transfection reagent (Thermo Fisher Sxientific, USA) according to the manufacturer’s instructions.

For in vivo studies, short hairpin RNAs (shRNAs) were applied and expressed using the pGPU6/GFP/Neo vector (GenePharma, Shanghai, China) for stably knockdown of LINC01016 in HGC27.

### Fluorescent in Situ Hybridization (FISH)

To detect the location of LINC01016, FISH was conducted with Ribo Fluorescent in Situ Hybridization Kit (RiboBio, Guangzhou, China). After fixation and permeation, pre-hybridization BC cells were incubated with cy3-labeled probe against LINC01016, U6 snRNA and 18 s rRNA at 37 °C overnight. Washing repeatedly, cells were stained with DAPI. Positive control probes U6 and 18 s (nuclear and cytoplasmic components) were set. Images were obtained with a fluorescence microscope (Olympus, Tokyo, Japan).

### Subcellular fractionation location

Separation of the nuclear and cytosolic fractions was carried out with the PARIS Kit (Invitrogen, Life Technologies Co, CA, USA). Briefly, the cells were collected and lysed on the ice, followed by centrifugation at 12,000 g for 3 min. The supernatant was analyzed for the cytoplasmic RNA and the nuclear pellet was used for detection of the nuclear RNA. For quantification PCR, U6 was used as nuclear control and GAPDH served as cytoplasmic control.

### Quantitative real-time polymerase chain reaction (qRT-PCR)

Total RNAs were prepared from the tissues and cells using TRIzol (Invitrogen). Complementary DNA (cDNA) from 1 μg total RNA was synthesized with a reverse transcriptase cDNA synthesis kit (Toyobo, Osaka, Japan), and the amplification reaction was conducted using FastStart Essential DNA Green Master (Roche Diagnostic GmbH, Mannheim, Germany). The relative expression of LINC01016 to GADPH was assessed using the 2^−ΔCT^ method.

### Colony formation assay

For colony formation assay, GC cells were planted and incubated into 6-well plates with 1000 cells/well at a humidified atmosphere for 14 days. The medium was changed every 2-3 days. Then, the colonies were fixed in formalin and stained with crystal violet, and finally calculated.

### Cell proliferation, wound healing and Transwell^®^ assays

Colony formation assay, MTS and EDU proliferation assay, Transwell assays were carried out as previously described [6].

### Flow cytometry analysis of the cell cycle and apoptosis

GC cells transfected with PCDNA3.1-LINC01016 or si-LINC01016 at a density of 2×10^5^ cells/well were cultured in 6 well plates for 48 h. Approximately 10^6^ cells were harvested, fixed, and resuspended in solution containing 500 μl PBS and 20 μl RNase (Beyotime). After 30 minutes of water bath at 37 °C, 400 μl propidium iodide (PI) was added to the cell suspension for cell cycle distribution analysis. The percentage of cells at each phase of the cell cycle was analyzed using Modfit 5.0. For cell apoptosis assay, the prepared cells were stained using an FITC Annexin V Apoptosis Detection kit (Beyotime, Shanghai, China) according to the manufacturer’s recommendations. Harvested cells were analyzed by flow cytometry (Beckman CytoFLEX FCM, Beckman Coulter, CA, USA) with CytExpert software.

### Western blot

Antibodies were purchased from three manufacturers: Abcam (SP-1, [ab231778]), Proteintech (EIF4A3 [17504-1-AP], MMP9 [82854-8-RR]). An anti-GADPH antibody (Bioss [bsm-33033M]) mainly was used as a loading control. The dilution of primary antibodies was 1:800-1:2000. Western blot analysis was performed as described previously [9].

### Immunohistochemistry

A streptavidin-peroxidase (S-P) approach was used to stain sections. Monoclonal rabbit anti-ki67 (1:500, Proteintech) was used to stain cells overnight at 4°C. Staining intensity was scored (0 = negative, 1 = weak, 2 = moderate, and 3 = strong), and the percentage of positively stained cells was determined (0 = 0%, 1 = 1–25%, 2 = 26–50%, 3 = 51–75%, and 4 = 76–100%). The IHC score was calculated using the equation: IHC score = P1 × 1 + P2 × 2 + P3 × 3 (P: percentage). These scores were added to produce the final score: high (score ≥ 4) and low or none (score = 0–3).

### Bioinformatics analysis

The LINC01016 sequence was downloaded from UCSC Genome Browser (http://genome.ucsc.edu/), from which the 1,000-bp transcription start site (TSS) upstream sequence was extracted. To identify putative transcription factors, the promoter sequence of LINC01016 was submitted to the JASPAR program (http://jaspar.genereg.net/).

### Vector construction

The putative LINC01016 promoter regions (-1000/0, -750/0, -500/0, -250/0, and -125/0) were PCR-amplified from the genomic DNA of HGC27 cells, which were inserted into the KpnI-XhoI sites upstream of the firefly luciferase in the pGL3-Basic vector (Promega, Madison, WI, USA). All of the constructs were named

based on the location of the promoter fragments relative to the TSS. EIF4A3, KLF-5, SP-1, ETS-1 and MEIS-1 plasmids were purchased form Vigene Biosciences (Rockville, MD, USA). ELK1, FOS, JUN, EGR, SP-1,and E2F-1 plasmids were from in our previous study. CEBP-β plasmid was provided by Mr. Sun (Shandong University). All vectors were confirmed by direct sequencing using primers shown in Supplementary Table 1.

### Luciferase assay

HGC27 and BGC823 were plated into 24-well plates at a density of 10^5^ cells/well and then co-transfected with the transcription factor plasmid (0.5 μg), LINC01016 promoter-luciferase reporter plasmid (0.5 μg), and pRL-TK plasmid (0.01 μg) per well with tuborfect transfection reagent. pRL-TK was used as the internal control. Forty-eight hours after transfection, a Dual-Luciferase Reporter Assay System (Promega) was used to determine the luciferase activity according to the manufacturer’s instructions.

### RNA pull-down assay

Biotinylated full-length sense or antisense LINC01016 and LINC01016 truncation probes were synthesized with a MEGAscript T7 Transcription Kit (Ambion, Carlsbad, CA, USA) and purified using a MEGAclear Kit (Ambion). 3 μg biotinylated RNA oligomers were mixed with pre-cleared protein lysates (1 mg), incubated with 60 μl washed streptavidin beads (Invitrogen) with rotation for 1 h. Proteins which bound to sense or antisense LINC01016 were pulled down, separated using SDS-PAGE and the specific bands were identified using mass spectrometry. Candidate proteins were defined as those that were detectable with a minimum of two peptides in at least two experimental repeats in both GC cells, with at least 2.5-fold enrichment compared to the control antisense LINC01016 RNA pull down. At the same time, molecular weight, subcellular localization and function should also be considered in the screening of proteins.

### RNA immunoprecipitation (RIP) assay

RIP experiments were implemented with the Magna RIP RNA Binding Protein Immunoprecipitation Kit (Millipore, Burlington, MA, USA) according to the instructions. Briefly, approximately 2 × 10^7^ cells were harvested by scraping and resuspended in RIP lysis buffer and mixed with protein A/G beads and anti-SP-1 or IgG antibody. Finally, the co-precipitated RNAs were pulled down by magnetic beads, washed by RIP washing buffer, and then assayed by qRT-PCR.

### RNA-Seq Bioinformatic Analysis

The mRNA sequencing (mRNA-seq) experiments were performed by Annoroad (Beijing, China). The quality of each sample was assessed by Agilent 2100 RNA Nano 6000 Assay Kit (Agilent Technologies, CA, USA). The mRNA-seq library was prepared for sequencing using standard Illumina protocols. Briefly, total RNAs from pEnter- or pEnter-EIF4A3-transfected HGC27 cells were isolated using TRIzol reagent (Invitrogen). Raw Reads were sequenced by Illumina platform, and high quality Clean Reads were obtained through data processing by removing low quality sequences and eliminating joint contamination. All subsequent analyses were based on Clean Reads. The information analysis process is mainly divided into three parts: sequencing data quality control, data comparison analysis and transcriptome deep analysis. Uniquely localized reads were then used to calculate read numbers and FPKM (Fragments per Kilobase per Million Mapped Fragments) values for each gene according to reads and genomic location. After obtaining the expression level of all genes in all the samples, differentially expressed genes were analyzed by using edgeR [10]. The genes were selected according to the following criteria: 1) with an absolute log2 fold change of > 2 in EIF4A3-overexpressing cells than in the control group; 2) a false discovery rate of < 0.05 were considered differentially expressed; 3) It is related to migration and invasion; and 4) oncogenic function.

### Chromatin immunoprecipitation (ChIP) assay

ChIP assay was performed using the EZ-Magna Chromatin Immunoprecipitation Kit (Millipore, Darmstadt, Germany) following the instructions of manufacturer. In short, cells were incubated with formaldehyde for 10 minutes to form DNA protein cross-linking, quenched with 0.125 M glycine, centrifuged at 4°C for 5 min at 800 g, and then dissolved in the sodium dodecyl sulfonate buffer with a protease inhibitor mixture. The cross-linked cell lysates were sonically and immunoprecipitated with anti-SP-1 antibody (Abcam, Cambridge, England). Input is the positive control and IgG is the negative control. Antibody binding complexes were collected using protein A/G magnetic beads and immunoprecipitation was elution by chip-elution buffer.The enrichment of LINC01016 and EIF4A3 promoters was detected by qRT-PCR assay.

### Tumor xenograft model

Four-week-old female Nu/nu athymic nude mice was purchased from Weitonglihua Biotechnology (Beijing, China). HGC27 cells were transfected with LV-shLINC01016 or LV-NC, and then cells were injected into the right dorsal flanks (8×10^5^ cells) or the lateral tail vein(8×10^5^ cells). Each group had 5 nude mice. The nude mice were randomly assigned to the experimental group and the control group by simple randomization method. Tumor growth was observed weekly and tumor volume (V) was calculated as V = (tumor length × width^2^)/2. Seven weeks after injection, the mice were killed, and the tumor nodules were collected. The tumors, lungs, and liver were isolated from the mice for further analysis. For animal trials, we estimated the sample size by obtaining relevant information from pre-experiments or published articles, using a double-blind method for analysis. All experimental procedures were approved by the Committee on the Ethics of Animal Experiments of the Shandong University.

### Statistical analysis

Statistical analyses were conducted using SPSS 22.0 or GraphPad Prism software (version 5.0, USA). Clinicopathological characteristics were analyzed by chi-square tests. Survival curves were calculated using Kaplan–Meier and log-rank tests. The effects of variables on survival were determined by univariate and multivariate Cox proportional hazards modeling. For comparison of two groups, a two-tailed Student’s *t* test was used. Comparison of multiple groups were made by a one-way ANOVA. All data were presented as the mean ± standard deviation (SD), determined from three independent experiments. *P* values (two-sided) ≤ 0.05 were considered to indicate statistical significance.

## Results

### LINC01016 is upregulated in GC with lymph node metastasis

**Fig. 1:**
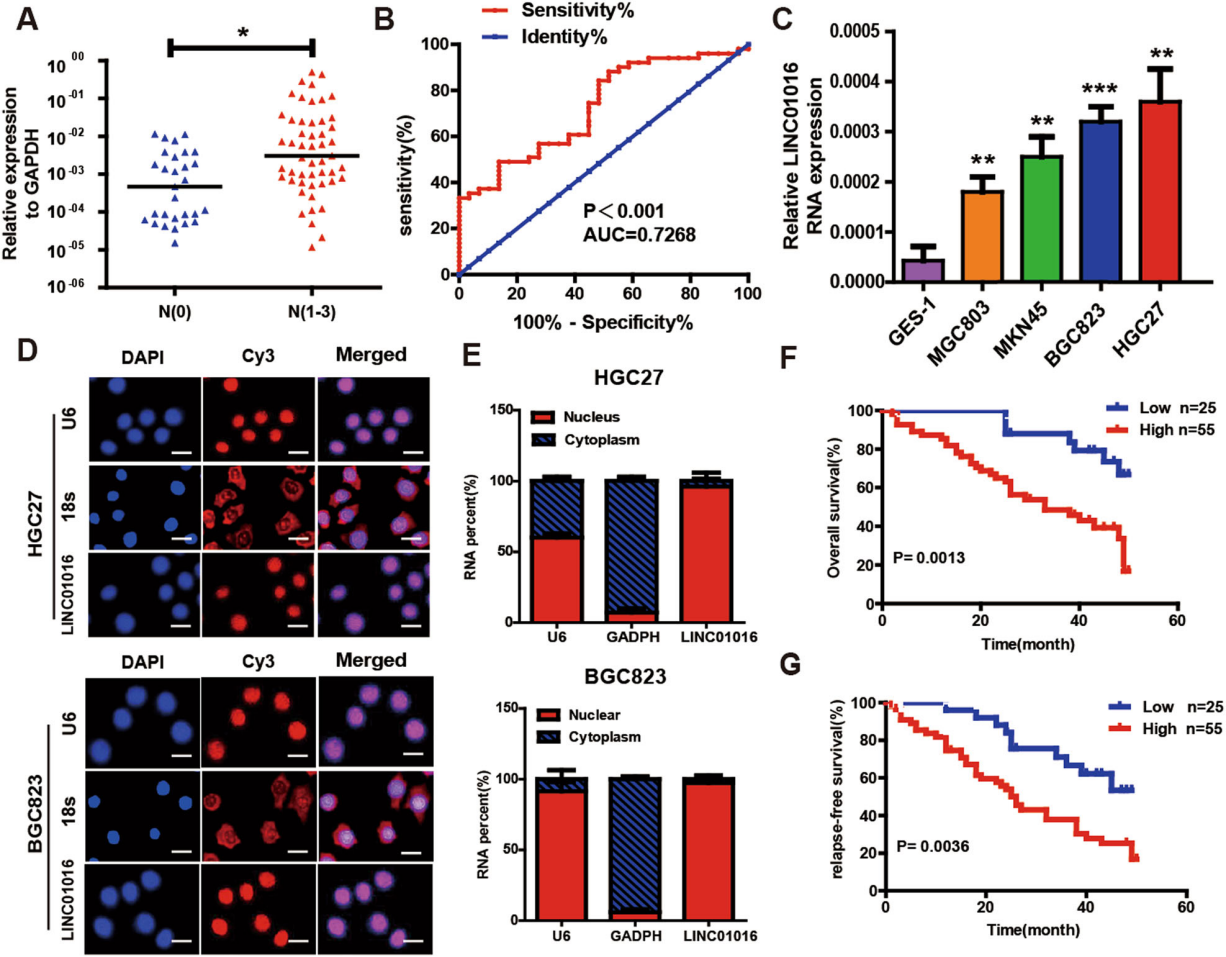
LINC01016 is upregulated in GC tissues with LNM and is associated with GC progression. **A** LINC01016 is highly expressed in GC tissues with LNM (*n* = 51) than in those without LNM (*n* = 29). **B** ROC curve is used to identify that LINC01016 could be used to distinguish the patients with LNM. **C** LINC01016 is notably upregulated in GC cell lines, especially in HGC27 and BGC823 cell lines. **D** RNA FISH assays reveals that LINC01016 is primarily located in the nucleus. U6 and 18S are utilized as controls for these localization analyses. **E** LINC01016, U6, and GAPDH levels are assessed in nuclear and cytoplasmic GC cell fractions by qRT-PCR. **F**–**G** GC patients in the high-LINC0106 group (*n* = 55) have shorter overall survival (OS) and relapse-free survival (RFS) than those in the low-LINC01016 group (*n* = 25). Higher than median LINC01016 expression is defined as high expression. On the contrary, the definition is low expression. Scale bar: 100μm. *P < 0.05, **P < 0.01,***P < 0.001.

First, differential gene expression analysis of lncRNAs was performed between five cases of gastric cancers with LNM and another five cases without LNM using the Human Gene Expression Microarray (Human 8×60 K LncRNA Microarray v2.0; Arraystar, Rockville, USA). Microarray data were uploaded to the NCBI Gene Expression Omnibus (GEO, GSE72307). The screening criteria and results have been published in Cancer Letters [11]. The gene expression profile evaluation showed that LINC01016 levels were significantly higher in GC tissues with LNM than in those without LNM (raw data: LNM: 6209.96 ± 12.43 & without LNM: 2251.38 ± 10.81, absolute fold-change = 3.07, *P* = 0.01). To determine whether LINC01016 participates in gastric metastasis, we collected and analyzed 80 samples of GC tissues with and without LNM. The qRT-PCR results indicated that the expression of LINC01016 was 20-fold higher in GC patients with LNM than in those without LNM (Fig. 1A). The receiver operating characteristic (ROC) curve suggested that LINC01016 could be used to distinguish patients with or without LNM, with the area under the curve reaching up to 0.7268 (95% confidence interval (CI) = 0.6146 to 0.8391, Fig. 1B).

Subsequently, evaluating the expression of LINC01016 in human normal gastric epithelial cell line GES-1 and gastric cancer cell lines HGC27, BGC823, MKN45, MGC803 revealed that LINC01016 was notably upregulated in GC cell lines compared with GES-1, especially HGC27 and BGC823 (Fig. 1C). FISH and qRT-PCR assays demonstrated considerable LINC01016 enrichment the nucleus (Fig. 1D, E).

The relationships between LINC01016 and clinicopathological features were also analyzed. High LINC01016 expression was associated with the degree of tumor differentiation, depth of invasion, and LNM (Table 1). As shown in the Kaplan Meier survival curve, GC patients with high-LINC0106 expression group had markedly shorter overall survival (OS) and relapse-free survival (RFS) than those in the low-LINC01016 group (Fig. 1F, G). Additionally, multiple factors associated with OS and RFS outcomes were evaluated using the univariate and multivariate cox regression models. The degree of tumor differentiation, LNM, and LINC01016 expression correlated with the survival outcomes of GC patients. The multivariate analysis showed that LNM is an independent prognostic factor for worse OS and RFS among GC patients (Table 2).

**Table 1:** Correlation between LINC01016 expression and patients’clinicopathological characteristics.

Character	Number	LINC01016 Expression		*P*
		High	Low	
Gender				
Male	59	44	15	0.0981
Femal	21	11	10	
Age				
≤60	40	23	17	0.0525
> 60	40	32	8	
differentiation				
Moderate	39	20	19	0.0015
Poor	41	35	6	
Lauren’s classification				
Intestinal	59	44	15	0.0981
Diffuse	21	11	10	
Tumer size(cm)				
≤4 cm	34	20	14	0.1431
> 4 cm	46	35	11	
Invasion depth				
T1-2	20	8	12	0.0023
T3-4	60	47	13	
Lymphatic metastasis				
N0	29	13	16	0.0009
N1-3	51	42	9

**Table 2:** Univariate and multivariate Cox proportional hazards regression analysis of overall survival and disease-free survival after surgery.

variable	univariate analysis			multivariate analysis		
	*P* value	HR	95% CI	*P* value	HR	95% CI
**overall survival**						
Age (< 60 years vs. ≥ 60 years)	0.254	0.687	0.360–1.310			
Gender (Female vs. Male)	0.899	0.954	0.463–1.966			
Differentiation (Moderate vs.Poor)	**0.021**	0.461	0.240–0.888	0.658	1.181	0.565– 2.471
Tumor size(> 4 cm vs. ≤4 cm)	0.14	1.626	0.853–3.098			
T stage (T1/2 vs. T3/4)	0.061	0.516	0.258–1.031			
N stage (N0 vs. N1/2/3)	**<0.0001**	0.231	0.119–0.447	**0.002**	0.200	0.072–0.545
Expression of LINC01016 (Low vs. High)	**0.001**	0.336	0.173–0.653	0.045	0.414	0.175–0.980
**Disease-free survival**						
Age (< 60 years vs. ≥ 60 years)	0.065	0.578	0.323–1.035			
Gender (Female vs. Male)	0.337	0.733	0.389–1.382			
Differentiation (Moderate vs.Poor)	**0.002**	0.389	0.213–0.709	0.969	0.987	0.510–1.912
Tumor size(> 4 cm vs. ≤4 cm)	0.017	2.02	1.132–3.604	0.419	0.764	0.398–1.468
T stage (T1/2 vs. T3/4)	0.004	0.406	0.220–0.750	0.365	0.663	0.273–1.613
N stage (N0 vs. N1/2/3)	<0.0001*	0.141	0.077–0.258	**0.000**	0.100	0.036–0.281
Expression of LINC01016 (Low vs. High)	**0.004**	0.417	0.231–0.752	0.422	0.738	0.351–1.550

Data that have statistical significance are highlighted in bold.

### SP-1 activates LINC01016 transcription and facilitates the migration and invasion of GC cells

**Fig. 2:**
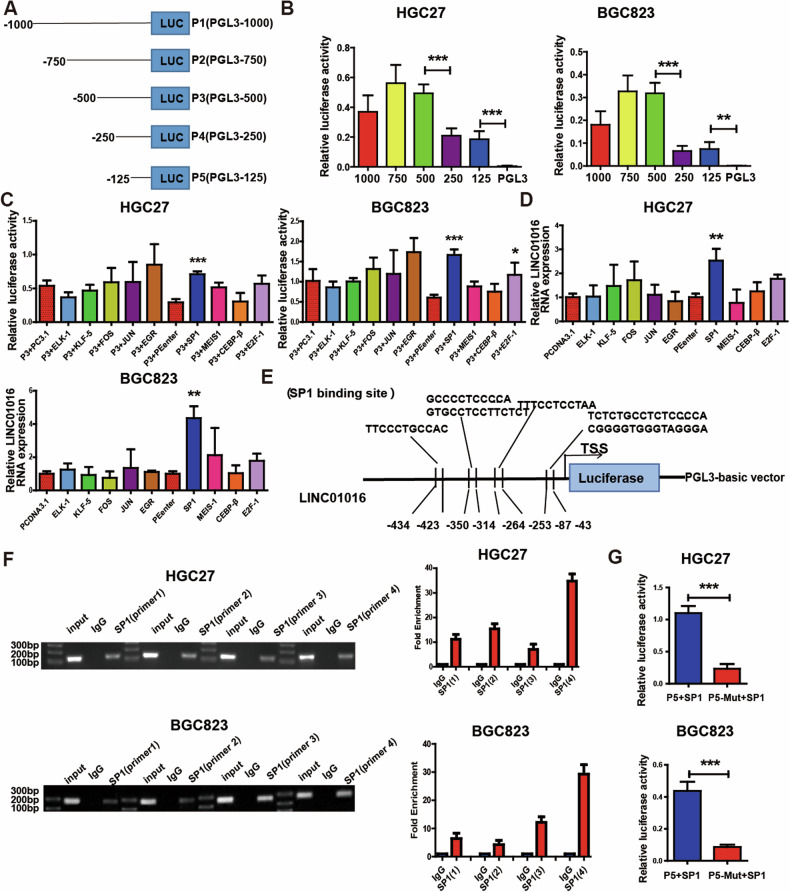
SP-1 activates LINC01016 transcription and facilitates the migration and invasion of GC cells. **A** Fragments of the LINC01016 promoter are cloned into the pGL3-basic vector upstream of firefly luciferase. (pGL3-1000, pGL3-750, pGL3-500, pGL3-250, pGL3-125) **B** Luciferase activity assays disclose that the promoter activity is significantly decreased from pGL3-500 to pGL3-250 and pGL3-125 to pGL3-0. **C** SP-1 and E2F-1 overexpression lead to a significant increase in luciferase activity. **D** qRT-PCR indicates that SP-1 overexpression promotes the expression of LINC01016 in GC cells. **E** The JASPAR website analyzes the potential SP-1 binding sites in the LINC01016 promoter (from −434 to −43 bp), divided into four regions. **F** Four pairs of primers are constructed to cover the SP-1 binding region, all of which could amplify PCR products from the immunoprecipitated DNA fragments of anti-SP-1 antibody, especially primer 4. **G** LINC01016 binding site mutants exhibite reduced luciferase activity. Data are presented as means ± SEM, from three independent experiments. *P < 0.05, **P < 0.01,***P < 0.001.

Recently mounting evidences have implied that transcription factors (TFs) played a decisive role in modulating the transcription of several lncRNAs [12, 13]. To explore the reason of LINC01016 overexpression, we obtained the possible binding sequence of LINC01016 transcription factors through the UCSC Genome Bioinformatics Site (http://genome.ucsc.edu/), and constructed multiple groups of truncated plasmids (Fig. 2A). A luciferase activity assay suggested that deletion of up to 250 bp resulted in an approximately 70% decrease in luciferase activity relative to that of PGL3-500/0 in HGC27 and BGC823 cells. Moreover, luciferase activity decreased when the −125 bp to 0 bp region was deleted. Thus, we believe the core promoter region of LINC01016 comprises two domains (−500 and −250 bp, −125 and 0 bp) (Fig. 2B).

Then the JASPAR algorithm was employed to analyze the candidate transcription regulators of LINC01016 in the transcription factor binding region from −500 to −250 bp and −125 to 0 bp (http://jaspar.genereg.net/downloads/) to determine the upstream regulatory mechanism of LINC01016. Nine molecules were screened out as potential transcription factors: ELK-1, KLF-5, FOS, JUN, EGR, SP-1, MEIS-1, CEBP-β, and E2F-1. However, only SP-1 enhanced LINC01016 expression in raising luciferase activity in the luciferase and qRT-PCR experiments (Fig. 2C, D). There were four possible binding regions of SP-1 based on the JASPAR website analysis, mainly between −434 and −43 bp base pairs (Fig. 2E). Four groups of primers were designed according to the four sections of the binding region for subsequent testing. A ChIP assay was used to determine whether SP-1 specifically interacts with the LINC01016 promoter. As shown in Fig. 2F, the PCR products amplified from the DNA fragments were immunoprecipitated by the anti-SP-1 antibody using primers covering the binding region of SP-1, particularly primer 4. The enrichment of the LINC01016 promoter amplicon by the anti-SP-1 antibody was ~30 times higher than that achieved with the anti-IgG antibody. To further confirm the direct interaction between SP-1 and the LINC01016 promoter, we generated deletion mutant constructs (P5-Mut) overexpressing SP-1, which resulted in a 70% reduction in luciferase activity in the P5-Mut group relative to the P5 wild type (Fig. 2G).

### LINC01016 enhances the migration and invasion capabilities of GC

**Fig. 3:**
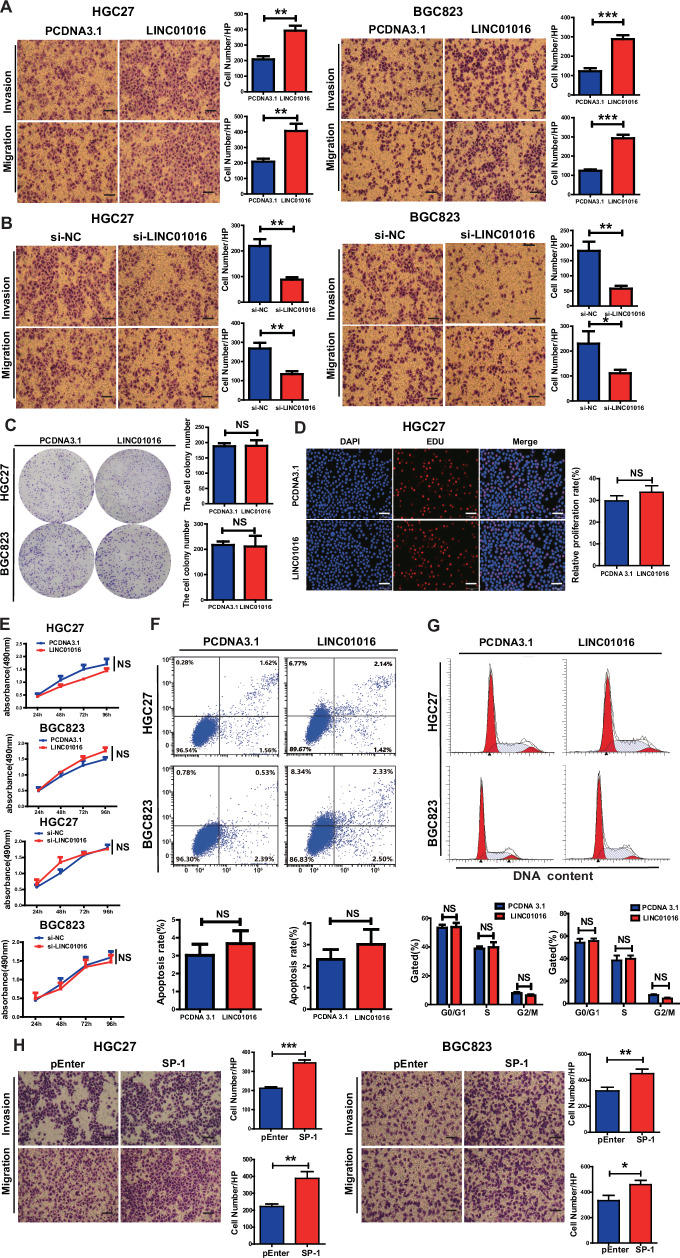
LINC01016 is associated with BC progression. **A**, **B** Transwell assays show that LINC01016 overexpression promoted cell migration and invasion, while konckdown this gene inhibited cell migration and invasion. **C**–**E** Colony formation EDU and MTS assays demonstrate that either overexpression or knockdown LINC01016 had no substantial influence on GC cell proliferation. **F**–**G** Flow cytometry assays show that LINC01016 did not affect on cell apoptosis or cell cycle distribution. **H** Transwell assays show that overexpression of ETS-1 enhances the ability of cell migration and invasion. Scale bar: 100 μm. Data are shown as mean ± SEM of three independent experiments, *P < 0.05, **P < 0.01,***P < 0.001, ns. not significant.

We overexpressed and knocked down LINC01016 expression by transfecting PCDNA3.1-LINC01016 and ASO into GC cells to clarify the effects of LINC01016 dysregulation in GC cells. The overexpression and knockdown efficiencies were determined by qRT-PCR (Supplementary Fig. 1A, B). Of the three tested ASO constructs, only si-LINC01016-3 successfully reduced LINC01016 expression by > 70%; thus, this construct was used in the subsequent functional studies (Supplementary Fig. 1B). Transwell assays revealed that the invasive and migratory capacities of GC cells were increased when LINC01016 was overexpressed, whereas silencing of LINC01016 resulted in opposite effects (Fig. 3A, B).

Additionally, proliferation tests were conducted to determine the effects of LINC01016, including colony formation, EDU, and MTS assays. However, the results demonstrated that either overexpression or knockdown LINC01016 had no substantial influence on GC cell proliferation (Fig. 3C, D, and E; Supplementary Fig. 1D, E, F, and G). The flow cytometry assays revealed that LINC01016 did not affect cell apoptosis or cell cycle distribution (Fig. 3F, G; Supplementary Fig. 1H, I).

To determine whether the effect of SP-1 on the biological behavior of GC cells is consistent with that of LINC01016, HGC27 cells and BGC823 cells were instantaneously transfected with pEnter and pEnter-SP-1. The transwell assay indicated that SP-1 overexpression could enhance migration and invasion in GC cell lines (Fig. 3H). To demonstrate that SP-1 promotes gastric cancer cell migration and invasion through the upregulation of LINC01016, a reversal experiment was conducted. The results indicated that, compared to the control group, overexpression of SP-1 with simultaneous knockdown of LINC01016 did not result in significant changes in cell migration and invasion. However, knockdown of SP-1 alongside LINC01016 overexpression significantly enhanced cell migration and invasion (Supplementary Fig.3). These findings suggest that SP-1 may promote gastric cancer cell migration and invasion via the upregulation of LINC01016.

### EIF4A3 is identified as a binding partner of LINC01016

**Fig. 4:**
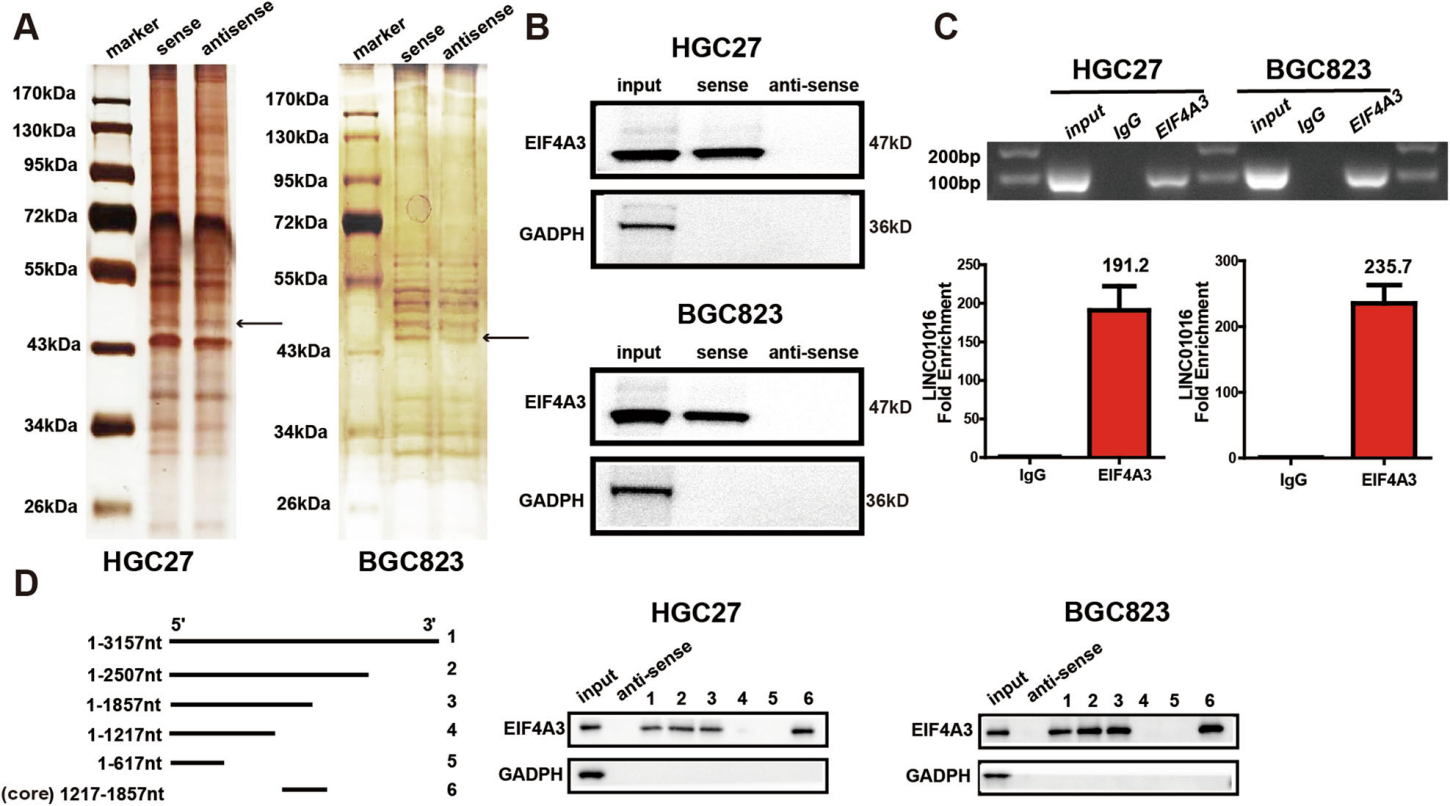
EIF4A3 is a nuclear binding protein of LINC01016. **A** A number of proteins in the range of 43–55 KDa that were co-precipitated by the LINC01016 sense transcript but not by the LINC01016 antisense transcript via pull-down assay and silver staining. **B** EIF4A3 is ultimately identified as a LINC01016-interacting protein by western blotting. **C** The binding of LINC01016 and EIF4A3 is confirmed via RIP assay, with qRT-PCR products being verified via agarose electrophoresis. **D** Truncated biotin-linked LINC01016 are used to pull down cellular protein and reveal that 1217–1857 nucleotide (nt) region could bind to EIF4A3. *P < 0.05, **P < 0.01,***P < 0.001.

LncRNA often affects cell function by competing for endogenous RNA (ceRNA) or binding proteins. Reportedly, LINC01016 affects cell function through a ceRNA mechanism; thus, we investigated whether it could play a role through binding to proteins. The proteins binding to LINC01016 was identified by an RNA pull-down experiment. We found several abnormal proteins in the range of 43-55 KDa that were co-precipitated by the LINC01016 sense transcript but not by the LINC01016 antisense transcript (Fig. 4A). Based on the MS results, two molecules, namely, eukaryotic initiation factor 4A-3 (EIF4A3) and HNRNPA3, were screened as candidate proteins (Supplementary Table 2). EIF4A3 was ultimately validated as the potential LINC01016-interacting protein by western blot (Fig. 4B). This result was independently confirmed via a RIP assay (Fig. 4C). Subsequently, RNA pull-down assays were conducted with different LINC01016 segment probes, and we found that only probes containing the 1217-1857 nucleotide (nt) region could pull down EIF4A3, suggesting that this is the core region for binding between LINC01016 and EIF4A3 (Fig. 4D).

### EIF4A3 reduces the levels of MMP9 mRNA and protein expression

**Fig. 5:**
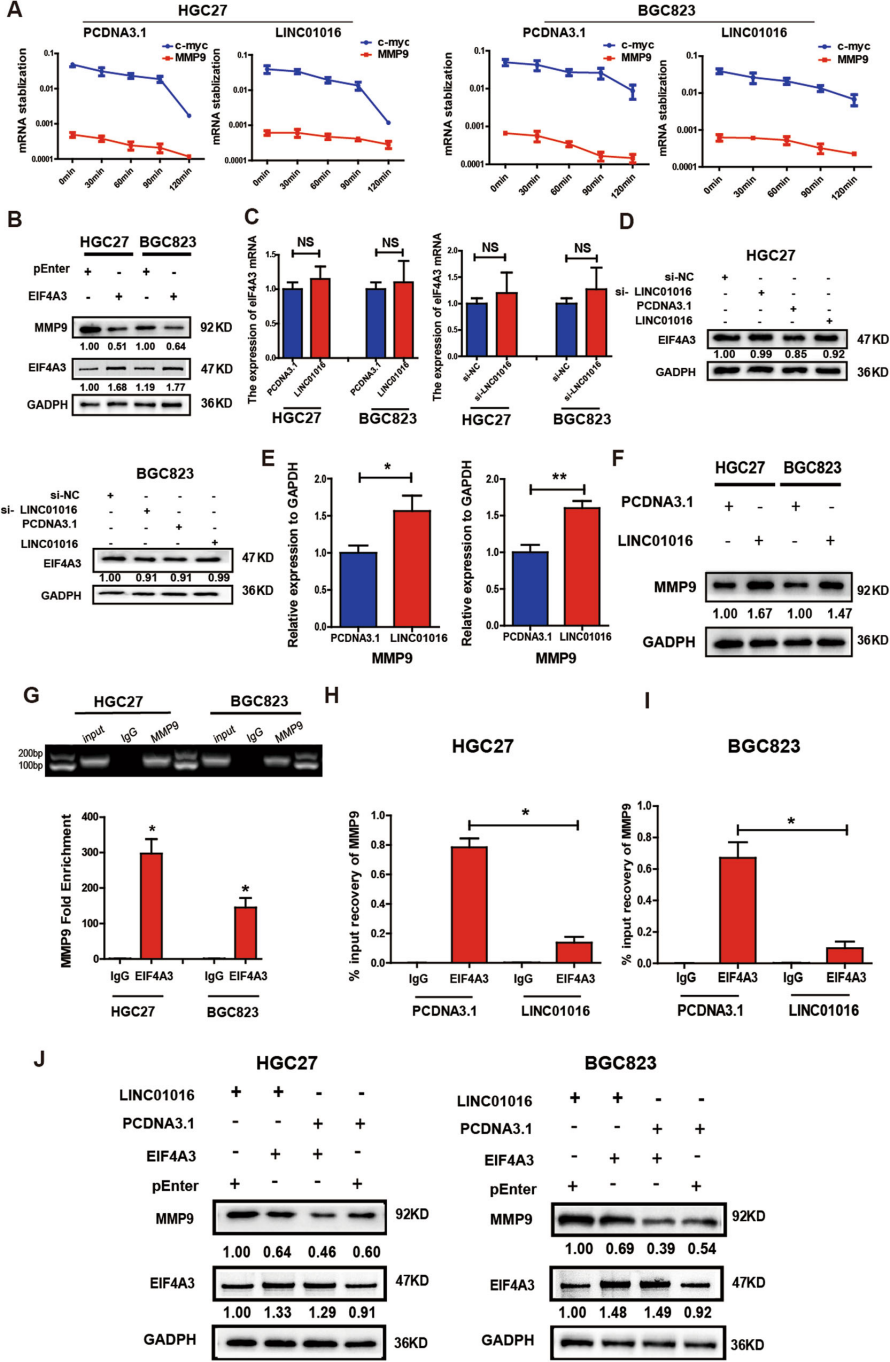
EIF4A3 reduces the level of MMP9 mRNA via NMD. **A** MMP9 mRNA stability was assessed via qRT-PCR in cells knockdown of LINC01016 and treated with Actinomycin D (20 μM) for 0, 30, 60, 90 and 120 min. Overexpression of LINC01016 had no effect on MMP9 mRNA expression levels. **B** EIF4A3 overexpression could reduce the protein level of MMP9. **C** Overexpression or knockdown of LINC01016 don’t change the mRNA level of EIF4A3. **D** Overexpression or knockdown of LINC01016 have no effect on EIF4A3 protein level. **E** When LINC01016 is overexpressed, the mRNA level of MMP9 is increased. **F** Overexpression of LINC01016 increase the MMP9 protein level. **G** RIP experiment shows that EIF4A3 could combine with MMP9 mRNA. **H**, **I** When LINC01016 is overexpressed, MMP9 mRNA binding to EIF4A3 is significantly decreased. **J** LINC01016 promotes the expression of MMP9 protein, but when EIF4A3 is overexpressed, the increase of MMP9 protein level can be reversed. Data are shown as mean ± SEM of three independent experiments, *P < 0.05, **P < 0.01,***P < 0.001, *ns*. not significant.

EIF4A3 is an Asp-Glu-Ala-Asp (DEAD) box-family adenosine triphosphate (ATP)-dependent RNA helicase, and participates in the exon junction complex (EJC) [14, 15]. One of the critical cellular functions of the EJC is an RNA surveillance mechanism termed nonsense-mediated RNA decay (NMD), a quality control system that degrades mRNAs and prevents the accumulation of abnormal RNAs [16, 17]. Next, we investigated the mRNAs affected by EIF4A3 that are likely involved in GC cell migration and invasion. RNA-seq was performed to obtain the transcriptional profiles of HGC27 cells overexpressing EIF4A3. MMP9 mRNA levels in EIF4A3-overexpressing cells were more than 50% lower than in the control group (Supplementary Table 3). Combined with the screening criteria and the gene set enrichment analysis (GSEA) results of RNA-seq data, MMP9 may be an EIF4A3-affected molecule. Actinomycin D (ActD) Chase assay assessed MMP9 mRNA stability. MMP9 and c-myc RNA stability was then analyzed by qPCR. The results indicate that the level of MMP9 mRNA decreases over time. However, the decrease in MMP9 mRNA expression is less pronounced in the group overexpressing LINC010106 compared to the PCDNA 3.1 group (Fig. 5A). Then, we conducted western blotting and found that EIF4A3 overexpression could reduce the protein level of MMP9 (Fig. 5B).

### LINC01016 increases the levels of MMP9 mRNA and protein expression

We speculated that LINC01016 may increase the expression of MMP9 through EIF4A3 and played a tumor-promoting role. When LINC01016 was silenced or overexpressed, EIF4A3 mRNA and protein levels were not significantly altered (Fig. 5C, D). Next, the effects of LINC01016 on MMP9 mRNA and protein were further analyzed. qRT-PCR and western blotting results showed that the MMP9 mRNA and protein were significantly increased after LINC01016 was overexpressed (Fig. 5E, F).

### LINC01016 combines with EIF4A3, and weaks the NMD effect of MMP9 mediated by EIF4A3

How do LINC01016 and EIF4A3 affect MMP9 mRNA and protein expression? EIF4A3 is a core component of the EJC, which is involved in splicing, transport, translation, and NMD. Of these functions, the most widely studied and significant is NMD, which is the translation-dependent surveillance mechanism that recognizes mRNAs containing premature termination codons (PTC) to prevent the accumulation of abnormal mRNA and truncated proteins. We hypothesized that EIF4A3 could bind to MMP9 mRNA and act via nonsense-mediated RNA decay. The starBase v2.0 site predicts that EIF4A3 can bind to MMP9 mRNA, which decodes miRNA-ceRNA, miRNA-ncRNA and protein-RNA interaction networks from large-scale CLIP-Seq data (http://starbase.sysu.edu.cn/). We further demonstrated such interactions independently using a RIP assay employing anti-EIF4A3 in GC cell lysate samples. Finally, MMP9 was detected in EIF4A3-immunoprecipitated RNAs by qRT-PCR (Fig. 5G). We also found that when LINC01016 was overexpressed, MMP9 mRNA binding to EIF4A3 was impaired due to the binding of LINC01016 to EIF4A3 (Fig. 5H, I). Thus, LINC01016 promotes the expression of MMP9 protein, but when EIF4A3 is overexpressed, the increase in MMP9 protein can be reversed (Fig. 5J). The increase in MMP9 expression after LINC01016 overexpression may be related to decreased NMD, mediated by EIF4A3, increasing MMP9 mRNA and protein expression.

### EIF4A3 can potentially weaken the tumour-promoting effect of LINC01016

Before investigating the relationship between EIF4A3 and LINC01016 in the progression of gastric cancer, we first evaluated the role of EIF4A3 in this carcinogenic context. Overexpressing EIF4A3 significantly hampered GC cell migration and invasion capabilities in the transwell assay (Supplementary Fig. 2A, B), and knocking this gene down had the opposite impact (Supplementary Fig. 2C, D). This finding suggests that EIF4A3 may negatively regulate malignant tumor behavior. Rescue experiments were performed to assess whether EIF4A3 was involved in the LINC01016-mediated reduced GC cell migration and invasion. HGC27 and BGC823 cells were co-transfected with LINC01016 and EIF4A3 plasmids. As displayed in Supplementary Fig. 2E, F, EIF4A3 attenuated the cell migration and invasion activity induced by LINC01016.

### LINC01016 may be a potential therapeutic target in GC

**Fig. 6:**
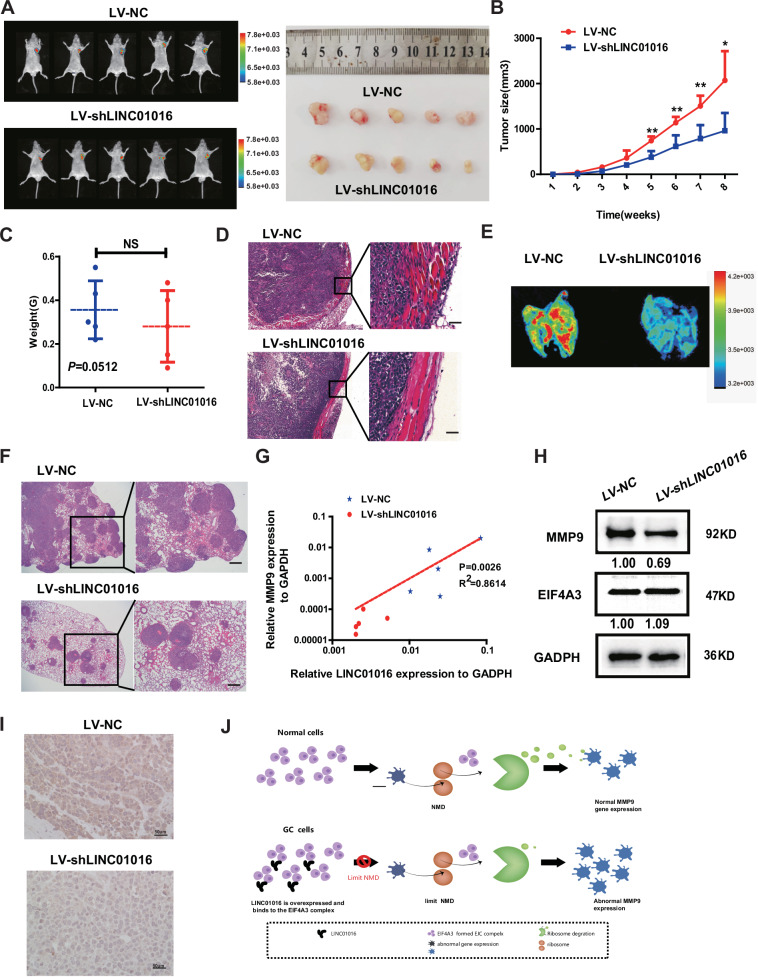
LINC01016 may be a potential therapeutic target in GC. **A**, **B** The tumor volume of the LV-NC group was slightly larger than that of the LV-shLINC01016 group. **C** The tumor weight in the LV-NC group was slightly higher than that in the control group, but the results were not statistically significant. **D** Tumors from mice in the LV- shLINC01016 group were well encapsulated in fibrotic capsules, whereas those from mice in the LV-NC group exhibited local muscular invasion (magnification: 200×). **E**, **F** The LV-NC group had more lung metastases than the LV-shLINC01016 group, after injecting the transfected cell to the tail vein of the nude mice. **G**, **H** The mRNA and protein levels of MMP9 were significantly lower in the LV-shLINC01016 group than in the LV-NC group. **I** Immunohistochemistry was used to detect MMP9, verifying that tumor cells in the LV-shLINC01016 group exhibited a lower positivity rate than that in the LV-NC group (magnification: 40×). **J** LINC01016 or LINC01016-mediated EIF4A3/MMP9 regulates the migration and invasion ability of GC cells. Scale bar: 100μm. Data are shown as mean ± SEM of three independent experiments, *P < 0.05, **P < 0.01,***P < 0.001, *ns*. not significant.

To elucidate whether LINC01016 could affect tumor growth and metastasis in vivo, we injected LINC01016 stable-knockdown HGC27 cells into the subcutaneous and caudal veins of male nude mice to construct a xenograft tumor model. The knockdown efficiency of LINC01016 was tested by qRT-PCR (Supplementary Fig. 1C). Eight weeks later, the nude mice were sacrificed (Fig. 6A). Although the tumor volume of LV-NC group was slightly larger than that of the LV-shLINC01016 group after the fifth week (Fig. 6B), no significant tumor quality differences were observed (Fig. 6C). We also observed that subcutaneous xenograft tumors in the LV-shLINC01016 group were non-invasive or well encapsulated, whereas the tumors in the LV-NC group invaded locally into muscular tissue (Fig. 6D). Using a hematogenous metastasis model, in vivo imaging revealed that the LV-shLINC01016 group presented fewer pulmonary metastatic lesions than the LV-NC group (Fig. 6E). The same conclusion was obtained via H&E staining (Fig. 6F). These results strongly suggest that LINC01016 plays a vital role in enhancing GC cell invasion and metastasis.

Finally, we explored the effect of knocking down LINC01016 on MMP9 mRNA and protein expression in xenograft tumors. The results showed that the mRNA and protein levels of MMP9 were significantly lower in the LV-shLINC01016 group than in the LV-NC group (Fig. 6G, H). Immunohistochemistry of MMP9 showed that the staining intensity of GC cells in the LV-shLINC01016 group was weaker than that in the LV-NC group (Fig. 6I). LINC01016 blocks the binding of EIF4A3 to MMP9 mRNA, thereby inhibiting EIF4A3-mediated NMD, leading to increased MMP9 mRNA and protein expression, and promoting tumor progression (Fig. 6J). LINC01016 or LINC01016-mediated EIF4A3/MMP9 may be a feasible target for GC patients.

## Discussion

Mounting evidence has suggested that lncRNAs are involved in the carcinogenesis and deterioration of diverse cancers through modulating chromatin architecture, genomic imprinting, and pre-/post-transcriptional regulation [18–21]. However, the concrete oncogenic mechanism of individual lncRNAs must be delineated in GC. In the present study, a lncRNA microarray analysis of GC revealed LINC01016 as a potentially oncogenic regulator of GC metastasis. LINC01016 was upregulated in GC samples with LNM. The upregulated LINC01016 level was related to the degree of tissue differentiation, T stage, LNM positivity status, and short RFS and OS. In addition, the multivariate analysis showed that LINC01016 could be an independent prognostic factor for GC.

LINC01016 is found to be located on chromosome 6q21.31, assembles with a transcript length of 3165 bp, and is frequently amplified in tumor tissues. Yun et al. analyzed LINC01016 acted as an oncogene and associated with thyroid carcinoma lymph node metastasis [22]. Philip et al. found that LINC01016 was the direct transcriptional target of the estrogen receptor (ER) and had prognostic significance for breast cancer patient survival [23]. While LINC01016 is considered an indicator of poor prognosis in breast and thyroid cancer, the mechanism remains unknown. Additionally, Xin et al. presented that LINC01016 by acting as a ceRNA of mir-302a-3p/mir-3130-3p to promote endometrial cancer disease progression [24]. However, the molecular mechanism by which LINC01016 is upregulated in these cancers has not been elucidated. This study is the first to explore the upstream mechanism of abnormal LINC01016 expression. We found that SP-1, as an upstream regulator, can enhance the transcription and expression of LINC01016 and significantly promote cell migration and invasion.

The biological functions of lncRNAs are closely related to their subcellular localization, cytoplasmic lncRNAs may be involved in regulating the stability and translation of mRNA [25, 26], while a nuclear-enriched lncRNA typically participates in transcriptional regulation by binding to nucleoprotein [27]. Liu et al. verified that lncNB1 bound to the ribosomal protein RPL35, leading to DEPDC1B gene transcription and subsequent regulation of ERK and N-Myc protein stabilization [28]. LINC01016 has been reported to promote cell migration and invasion in endometrial carcinoma by acting as a sponge absorber, similar to ceRNA. Whether LINC01016 can participate in tumor biological behavior through binding protein has not been reported. In this study, we found that LINC01016 was primarily located in the GC cell nuclei, and its 1217–1857 region physically combined with EIF4A3 nuclear protein. LINC01016 could affects downstream protein expression by binding to EIF4A3 and promotes the progression of gastric cancer.

The core of a larger complex called the exon junction complex (EJC) comprises EIF4A3 and three other proteins [29–31]. Within this core, the protein EIF4A3 collaborates with its binding partner MLN51 to function as the primary RNA binding constituent [32]. The EIF4A3-formed EJC has the ability to induce nonsense-mediated mRNA decay and can also regulate protein expression at the translational and post-translational levels [33]. Giorgi and colleagues demonstrated that knocking down EIF4A3 significantly increased both synaptic strength and GLUR1 AMPA receptor abundance at synapses [34]. Based on these findings, it is hypothesized that LINC01016 binds to EIF4A3 and influences its abundance in the mRNA region of MMP9, thereby impacting invasion and metastasis of GC.

In this study, we first detected the expression of MMP9 was significantly decreased in cells that were transfected with EIF4A3 compared to those transfected with pEnter. Further validation of the underlying mechanisms was performed by examining the expression of MMP9 after transfection with LINC01016 and/or LINC01016 together with EIF4A3 or pEnter. EIF4A3 eliminated the promoting effect of LINC01016 on MMP9 protein expression. EIF4A3 is an important component of the EJC complex and plays a supervisory role in RNA through NMD. RNA pull-down and RIP assays proved that EIF4A3 can bind to both LINC01016 and MMP9 mRNA. When LINC01016 is overexpressed, the binding of EIF4A3 and MMP9 mRNA is blocked, and NMD is inhibited, leading to the upregulation of MMP9, thus promoting tumor progression. MMP-9 can degrade the extracellular matrix (ECM) components and has an important role in tumor invasion and metastasis [35, 36].

In summary, LINC01016 overexpression could serve as a poorer prognostic indicator in patients with GC. LINC01016 can bind to EIF4A3, which combinates MMP9 and inhibit the formation of MMP9 mRNA through NMD. When LINC01016 is up-regulated, the competitive combination between LINC01016 and EIF4A3 weakens the NMD effect on MMP9 mediated by EIF4A3, increasing MMP9 expression, and promoting the progression of GC. LINC01016 or LINC01016-mediated EIF4A3/MMP9 may thus be a candidate target for GC therapy.

## Supplementary information

Supplementary Figure

Sup Table 1 primers

Sup Table 2 EIF4A3

Sup Table 3 MMP9 OE_NC

## Data Availability

The data that support the findings of our study are present in the paper. The rest datasets used or analyzed during the current study are available from the corresponding author on reasonable request.
