# Supplementary material for: SP-1-activated LINC01016 overexpression promotes gastric cancer invasion and metastasis through inhibiting EIF4A3-mediated MMP9 mRNA decay
Source: Cell Death Dis. 2025 Jan 29;16(1):54. doi: 10.1038/s41419-024-07250-z (PMC11828860; doi:10.1038/s41419-024-07250-z)
Supplement: Supplementary file 1 — Supplementary Figure [file 41419_2024_7250_MOESM1_ESM.docx]

*Supplementary information*

**SP-1-activated LINC01016 overexpression promotes** **gastric cancer invasion and metastasis inhibiting EIF4A3-mediated MMP9 mRNA decay**


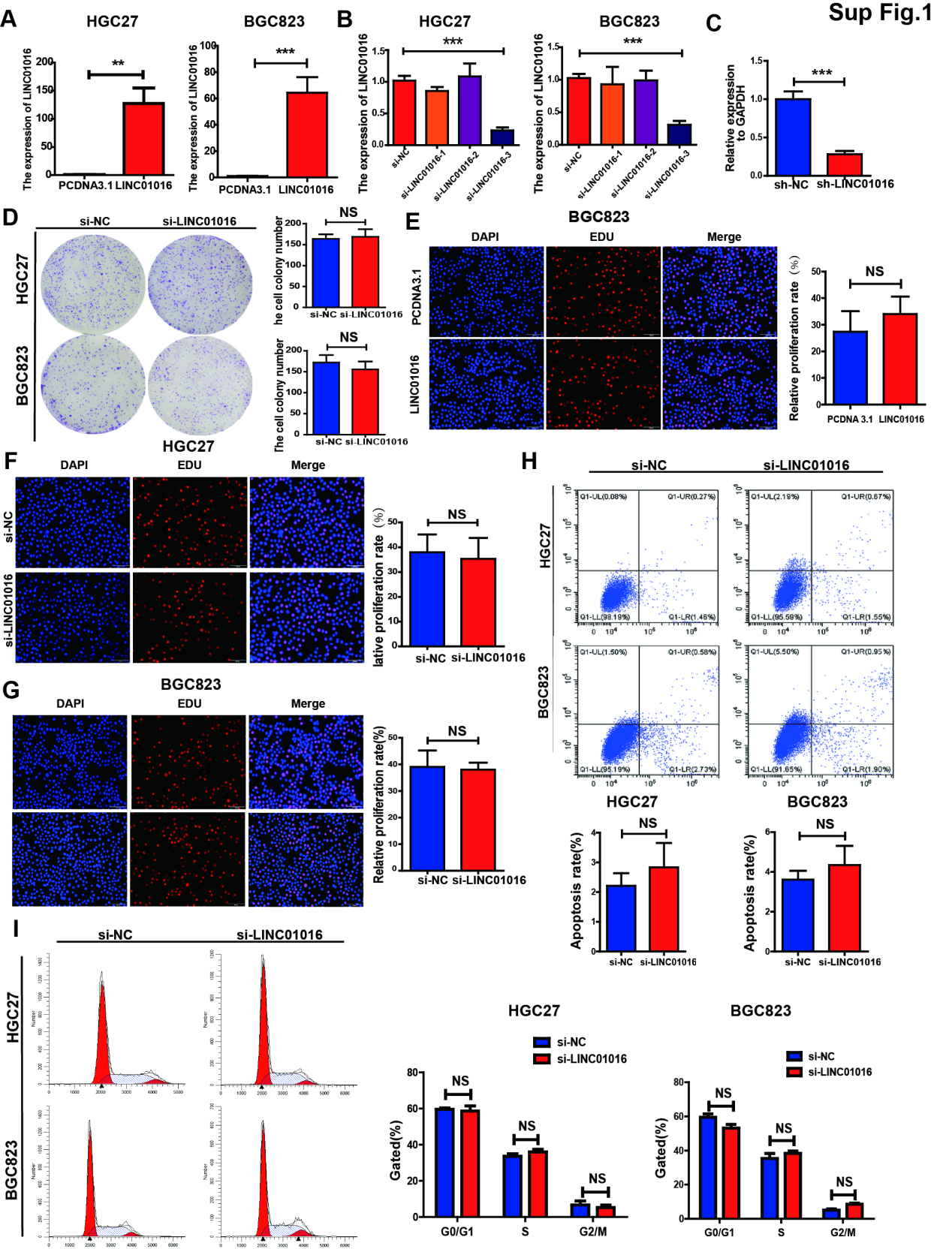


**Supplementary Fig. 1. LINC01016 has no significant effect on the proliferation and apoptosis of gastric cancer cells. A.** LINC01016 was overexpressed by transfection with PCDNA3.1-LINC01016 plasmid. **B.** Three different Antisense oligonucleotides (ASOs) specific for LINC01016 (si-LINC01016-1, si-LINC01016-2, and si-LINC01016-3) were obtained from RiboBio. Of the three tested ASO constructs, si-LINC01016-3 successfully reduced LINC01016 expression by > 70%. **C.** The qRT-PCR assay revealed that LINC01016 was stably knocked down in the LV-shLINC01016-transfected cells. **D.** Colony formation assays showed that knockdown of LINC01016 did not affect cell proliferation. **E-G.** EDU assays demonstrated that either overexpression or knockdown LINC01016 had no substantial influence on GC cell proliferation. **H-I.** Flow cytometry assays showed that LINC01016 did not affect on cell apoptosis or cell cycle distribution. Scale bar：100μm. Data are presented as means ± SD. **P* < 0.05, ***P* < 0.01,****P* < 0.001, *ns*. not significant.


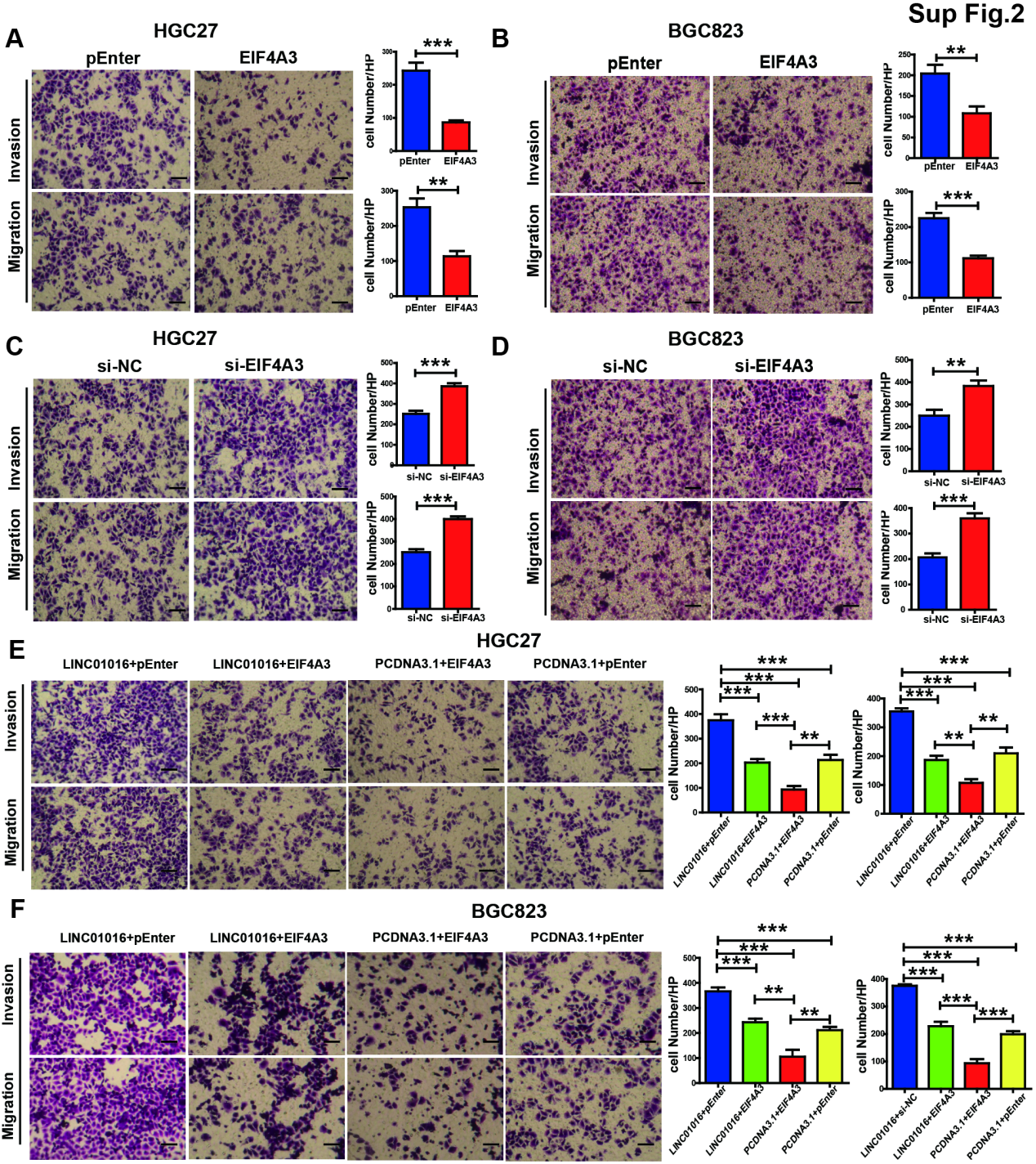


**Supplementary Fig. 2. EIF4A3 can potentially weaken the tumour-promoting effect of LINC01016 A-B.** Transwell assays show that EIF4A3 overpression inhibits cell migration and invasion. **C-D.** EIF4A3 knockdown promotes cell migration and invasion. **E-F.** EIF4A3 overexpression reverses the impact of LINC01016 overexpression on GC cell migration and invasion. Scale bar：100μm. Data are represent as the mean ± SEM from triplicate experiments. **P* < 0.05, ***P* < 0.01,****P* < 0.001, *ns*. not significant.


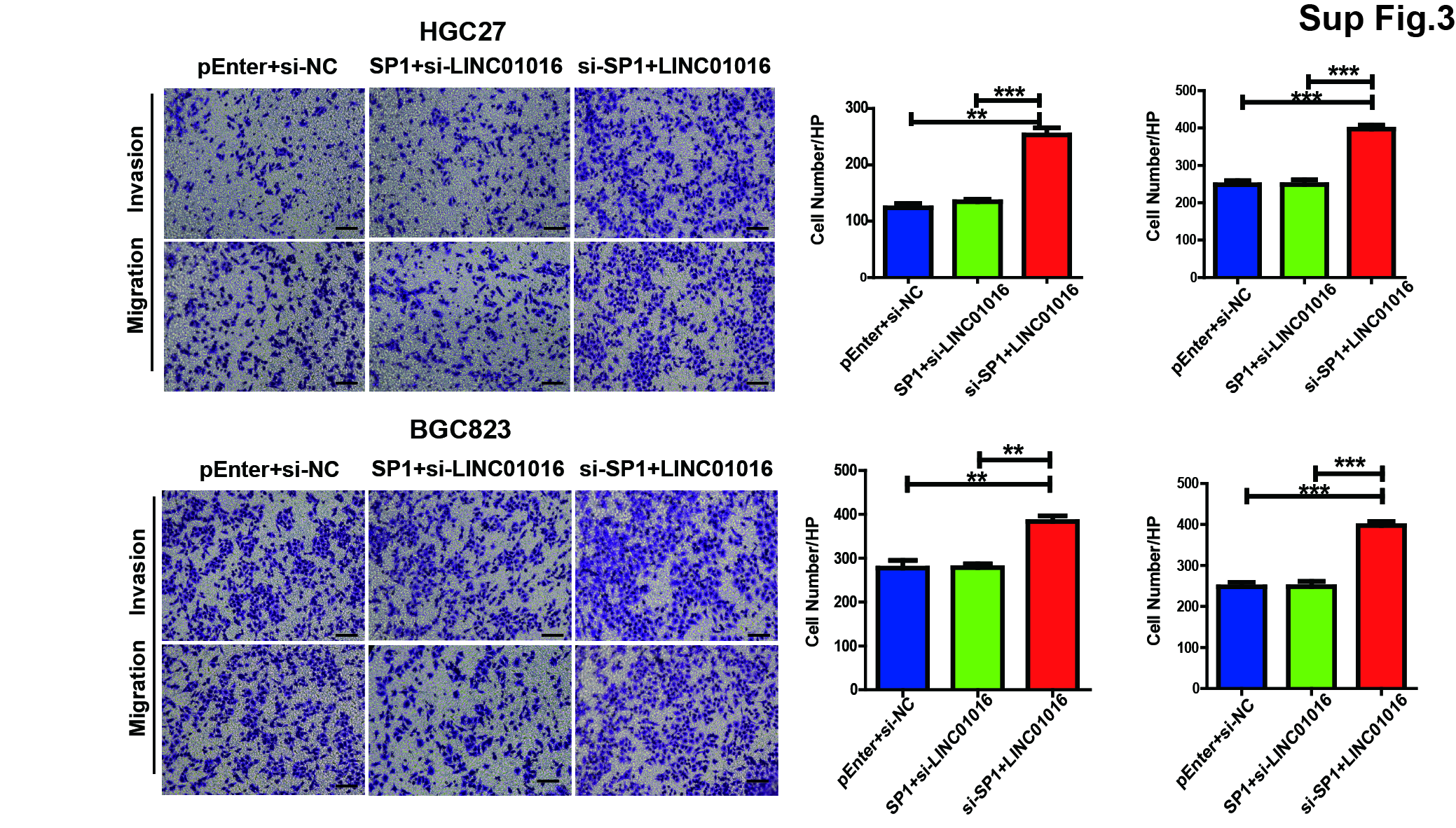


**Supplementary Fig. 3.** SP1 may promote gastric cancer cell migration and invasion via the upregulation of LINC01016.Transwell assays show that overexpression of SP-1 with simultaneous knockdown of LINC01016 did not result in significant changes in cell migration and invasion. However, knockdown of SP-1 alongside LINC01016 overexpression significantly enhanced cell migration and invasion. Scale bar：100μm. Data are represent as the mean ± SEM from triplicate experiments. *P < 0.05, **P < 0.01,***P < 0.001, ns. not significant.
